# Association between admission blood urea nitrogen and clinical outcomes in patients with large ischemic stroke undergoing endovascular treatment

**DOI:** 10.3389/fneur.2026.1880623

**Published:** 2026-09-23

**Authors:** Mingrui Zhou, Haisong Feng, Shuangzhi Wang, Kaiyuan Feng, Linyu Li, Lilan Wang, Haoxuan Zhu, Gaoming Li, Chunye Chen, Mingyang Chen, Yuhan Fan, Qiuyi Yang, Yihui Yang, Yian Chen, Binghan Wang, Guanting Heng, Xuexiao Zhao, Chawen Ding, Jiaxing Song, Miao Chai, Jie Pu

**Affiliations:** 1 The First Clinical Medical School, Hubei University of Traditional Chinese Medicine, Wuhan, Hubei, China; 2 Department of Encephalopathy, Hubei Provincial Hospital of Traditional Chinese Medicine, Wuhan, Hubei, China; 3 Department of Encephalopathy, Affiliated Hospital of Hubei University of Chinese Medicine, Wuhan, Hubei, China; 4 Hubei Key Laboratory of Theory and Application Research of Liver and Kidney in Traditional Chinese Medicine, Wuhan, Hubei, China; 5 Department of Neurology, The Third Hospital of Mianyang, Mianyang, Sichuan, China; 6 Department of Neurology, Xinqiao Hospital and The Second Affiliated Hospital, Army Medical University (Third Military Medical University), Chongqing, China; 7 Department of Neurology, The Second Affiliated Hospital of Chongqing Medical University, Chongqing, China; 8 Department of Neurology, The 908th Hospital of Chinese People’s Liberation Army Joint Logistic Support Force, Nanchang, China

**Keywords:** acute ischemic stroke, blood urea nitrogen, clinical outcomes, endovascular treatment, large ischemic stroke

## Abstract

**Background:**

This study aimed to investigate the association between admission blood urea nitrogen (BUN) and clinical outcomes in patients with large ischemic stroke undergoing endovascular treatment (EVT).

**Methods:**

This study was a subgroup analysis of a prospective, multicenter cohort study. 472 patients with large ischemic stroke undergoing EVT from the original database were included. Patients were stratified into three groups based on admission BUN levels. The primary effectiveness outcome was modified Rankin Scale (mRS) score 0–3 at 90 days. Safety outcomes included 90-day mortality and symptomatic intracranial hemorrhage (sICH) within 48 h.

**Results:**

A total of 472 patients were included and stratified into three groups: Tertile 1 group (≤5.00 mmol/L), Tertile 2 group (5.01–6.80 mmol/L), and Tertile 3 group (>6.80 mmol/L). Compared with patients in BUN Tertile 1 group, patients in BUN Tertile 3 had significantly lower odds of achieving a favorable outcome (adjusted odds ratio [aOR] 0.54, 95% CI 0.31–0.95, *p* = 0.032 in Adjusted Model 1), while BUN Tertile 2 group showed no statistical significance. There were no significant differences in mortality and sICH among three groups. Subgroup analysis presented a significant interaction between BUN and age for the primary effectiveness outcome (*P* for interaction = 0.036).

**Conclusion:**

In patients with large ischemic stroke undergoing EVT, an elevated admission BUN (>6.80 mmol/L) was associated with a lower likelihood of favorable functional outcome at 90 days.

## Introduction

Large ischemic stroke accounts for approximately 20% of patients in anterior circulation large vessel occlusion (LVO) stroke, contributing to the socioeconomic and healthcare burden (1, 2). Several randomized controlled trials (RCTs) have been conducted in recent years and confirmed that EVT offers greater efficacy and safety than standard medical treatment alone in the large ischemic stroke patients (3–8). Despite the established prognostic value of factors such as age, baseline neurological severity, extent of ischemic injury, collateral status, and reperfusion success, disability and mortality remain substantial among patients with large ischemic stroke undergoing EVT. Beyond these conventional predictors systemic physiological status may provide complementary prognostic information. Therefore, identifying readily available biomarkers that reflect systemic physiological disturbances may help provide a more comprehensive assessment of prognosis in this high-risk population.

Blood urea nitrogen (BUN) is an inexpensive and routinely available biochemical parameter that is influenced by multiple physiological conditions, including renal perfusion/function, hydration status, and protein catabolism. Several studies have demonstrated that an elevated BUN is associated with increased mortality in critically ill patients including patients with cardiovascular disease and patients admitted to intensive care units (9–11). Moreover, quite a few models predicting the prognosis of acute ischemic stroke (AIS) established in recent years utilized BUN as one of the independent factors (12–14). Furthermore, there has been a study indicated that higher BUN is significantly associated with an increased risk of all-cause in-hospital mortality in AIS patients (15). Nevertheless, these studies were limited to patients with small or medium core infarcts. The association between BUN and clinical outcomes in patients with large ischemic stroke undergoing EVT remained unclear.

Therefore, using data from a large, prospective cohort study (16), we aimed to evaluate the association between BUN and clinical outcomes in patients with large ischemic stroke undergoing EVT.

## Methods

### Study design and patient selection

We collected data from a prospective, multicenter cohort study which enrolled patients with large ischemic stroke across 38 comprehensive stroke centers in China between November 2021 and February 2023. The present analysis was restricted to patients undergoing EVT. Details of this registry have been described previously (16). Written informed consent was obtained from all participants or their legal representatives before enrollment. The initial study was carried out in accordance with the Declaration of Helsinki and was approved by the ethics committees of all participating centers.

Inclusion criteria were as follows: (1) age ≥18 years; (2) AIS due to anterior circulation LVO, defined as occlusion of the internal carotid artery (ICA) or the M1 or M2 segment of the middle cerebral artery (MCA); (3) within 24 h of stroke onset or last known well time; (4) large ischemic stroke, defined as an Alberta Stroke Program Early Computed Tomography Score (ASPECTS) of 0–5; (5) received EVT. The exclusion criteria for this study included: (1) patients with missing BUN data; (2) pre-stroke disability defined as modified Rankin Scale (mRS) score >2; (3) patients with severe or terminal illnesses; (4) lack of follow-up information at 90 days. Figure 1 gave an illustration of patient selection.

**Figure 1:**
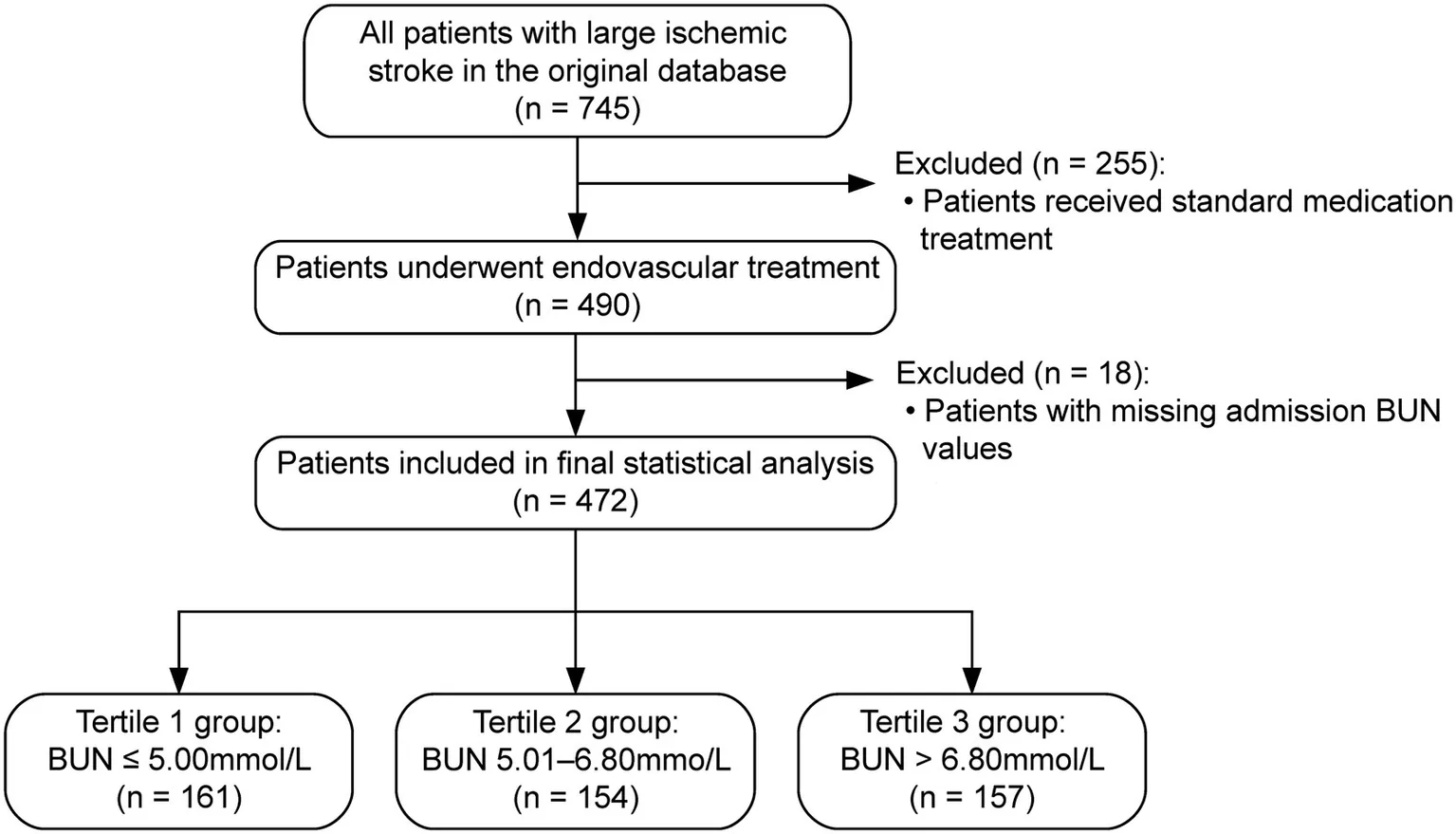
Flowchart of study participants selection.

### Data collection

Patients’ baseline characteristics were collected, including demographics, medical history, the severity of stroke, admission BUN and other laboratory data, stroke etiology, occlusion site, imaging characteristics, intravenous thrombolysis, workflow time (onset to imaging diagnosis, onset to puncture, onset to recanalization, and puncture to recanalization). Medical history included history of hypertension, diabetes mellitus, atrial fibrillation (AF), hyperlipidemia, and current or previous smoking status. The severity of stroke was defined with National Institutes of Health Stroke Scale (NIHSS) scores. Stroke etiology was classified in accordance with the Trial of ORG10172 in Acute Stroke Treatment (TOAST) classification. Imaging characteristics included the Alberta Stroke Program Early Computed Tomography Score (ASPECTS), the modified thrombolysis in cerebral infarction (mTICI) scale, with grades of 2b or 3 indicates successful recanalization after EVT, and collateral status based on the American Society of Interventional and Therapeutic Neuroradiology/Society of Interventional Radiology collateral grading system (ASITN/SIR).

### Outcome measures

The primary effectiveness outcome was a favorable functional outcome, defined as an mRS of 0–3 at 90 days. Secondary effectiveness outcomes included functional independence (defined as mRS of 0–2), the proportion of mRS 0–4, and ordinal score on mRS at 90 days. Safety outcomes included mortality within 90 days, symptomatic intracranial hemorrhage (sICH) within 48 h evaluated on follow-up CT or MRI according to the Heidelberg Bleeding Classification (17), and any intracranial hemorrhage within 48 h. The outcome measures in the present study were selected in accordance with the original MAGIC registry.

### Statistical analysis

Study participants were divided according to tertiles of BUN at admission: Tertile 1 group, BUN ≤5.00 mmol/L; Tertile 2 group, BUN 5.01–6.80 mmol/L; Tertile 3 group, BUN >6.80 mmol/L. The normality of distribution for continuous variables was assessed using the Kolmogorov–Smirnov test. As all continuous variables were found to be non-normally distributed, they are presented as median with interquartile range (IQR) and were compared across groups using the Kruskal-Wallis H test. Categorical variables are expressed as frequency and percentages (%) and were compared using the chi-squared or Fisher’s exact test.

The association between BUN tertiles and 90-day clinical outcomes was evaluated using multivariable logistic regression models. Specifically, binary logistic regression was applied to dichotomous outcomes including mRS 0–3, mRS 0–2, mRS 0–4, 90-day mortality, and sICH. The association with the ordinal 90-day mRS score was assessed using multivariable ordinal logistic regression, with the proportional odds assumption evaluated using the Brant test. To evaluate the association between BUN and clinical outcomes while controlling for potential confounding, three logistic regression models were constructed. The primary multivariable model (Adjusted Model 1) included age, sex, baseline NIHSS score, ASPECTS, occlusion site, stroke etiology, onset-to-recanalization time, and IVT, selected based on their established clinical relevance to functional outcome after EVT. In addition, an exploratory model (Adjusted Model 2), including all covariates in Model 1 except age, was constructed to evaluate the influence of age adjustment on the estimated association between BUN and clinical outcomes. The primary interpretation of the study was based on Adjusted Model 1. For each model, odds ratios (OR) and adjusted odds ratios (aORs), along with their 95% confidence interval (CI) were calculated with BUN Tertile 1 group designated as the reference group.

In addition, to account for potential clustering of patients within participating centers, sensitivity analyses were performed using mixed-effects logistic or ordinal logistic regression models, as appropriate. The covariates included in Adjusted Model 1 were entered as fixed effects, and participating center was included as a random intercept.

To minimize potential information loss resulting from categorization, admission BUN was additionally analyzed as a continuous variable (per 0.1 mmol/L increase) using multivariable logistic regression models. Marginal effect plots were generated to visualize the predicted probabilities of an mRS score of 0–3 at 90 days, an mRS score of 0–2 at 90 days, 90-day mortality, and sICH within 48 h across continuous BUN levels. Potential non-linear associations between BUN and these outcomes were further examined using restricted cubic spline (RCS) analyses with 5 knots. *p* values for the overall association and for non-linearity were reported. Both models were adjusted for the same covariates as Adjusted Model 1.

Exploratory subgroup analyses were conducted to assess potential effect modification according to age, sex, baseline NIHSS score, ASPECTS, stroke etiology, occlusion site, IVT, onset-to-puncture time, and ASITN/SIR grade. *p* values for interaction were calculated to assess heterogeneity across subgroups. Because these analyses were exploratory, no adjustment for multiple comparisons was performed.

For all statistical analyses, a two-sided *p* value <0.05 was considered to indicate statistical significance. Missing values are reported in Table 1. None of the covariates included in the multivariable regression models had missing data and no imputation was required for the regression analyses. Statistical analyses were performed using SPSS (version 27.0) and R software (version 4.4.1).

**Table 1:** Baseline characteristics of large ischemic stroke patients underwent EVT according to BUN stratification.

Characteristic	Overall	BUN tertile groups (mmol/L)			*p* value
		Tertile 1 (BUN ≤5.00)	Tertile 2 (BUN 5.01–6.80)	Tertile 3 (BUN >6.80)	
No. of patients	472	161	154	157	
Demographics characteristics					
Age (years), median (IQR)	69 (59–77)	66 (54–73)	68.50 (58–76)	74 (64–81)	<0.001
Male sex, *n* (%)	272 (57.6)	93 (57.8)	95 (61.7)	84 (53.5)	0.344
Medical history					
Hypertension, *n* (%)	288 (61.0)	98 (60.9)	96 (62.3)	94 (59.9)	0.904
Atrial fibrillation, *n* (%)	213 (45.1)	63 (39.1)	71 (46.1)	79 (50.3)	0.128
Diabetes mellitus, *n* (%)	72 (15.3)	23 (14.3)	29 (18.8)	20 (12.7)	0.300
Hyperlipidemia, *n* (%)	105 (22.2)	27 (16.8)	38 (24.7)	40 (25.5)	0.119
Smoking, *n* (%)	146 (30.9)	54 (33.5)	52 (33.8)	40 (25.5)	0.194
Clinical examination					
Baseline SBP (mmHg), median (IQR)^a^	146 (129–163)	146 (130–160)	147 (130–162)	143 (127–166)	0.931
Baseline DBP (mmHg), median (IQR)^a^	86 (76–96)	87 (77–97)	86 (74–94)	85 (76–96)	0.632
Baseline NIHSS score, median (IQR)	17 (14–20)	17 (13–20)	17 (14–21)	17 (14–21)	0.328
Glucose (mmol/L), median (IQR)^b^	7.15 (5.95–8.86)	6.90 (5.88–8.47)	7.27 (6.05–9.07)	7.40 (6.10–8.94)	0.497
Imaging characteristics					
ASPECTS score, median (IQR)	4 (2–5)	4 (2–5)	4 (2–5)	4 (2–5)	0.915
Occlusion site					0.119
ICA, *n* (%)	196 (41.5)	65 (40.4)	60 (39.0)	71 (45.2)	
M1, *n* (%)	226 (47.9)	86 (53.4)	73 (47.4)	67 (42.7)	
M2, *n* (%)	50 (10.6)	10 (6.2)	21 (13.6)	19 (12.1)	
ASITN/SIR grades					0.450
0 to 1, *n* (%)	228 (48.3)	73 (45.3)	77 (50.0)	78 (49.7)	
2, *n* (%)	165 (35.0)	58 (36.0)	48 (31.2)	59 (37.6)	
3–4, *n* (%)	79 (16.7)	30 (18.6)	29 (18.8)	20 (12.7)	
Stroke etiology					0.187
LAA, *n* (%)	142 (30.1)	51 (31.7)	51 (33.1)	40 (25.5)	
CE, *n* (%)	268 (56.8)	83 (51.6)	88 (57.1)	97 (61.8)	
Others, *n* (%)	62 (13.1)	27 (16.8)	15 (9.7)	20 (12.7)	
Procedure time, min, median (IQR)					
Onset to imaging	294 (162–455)	324 (192–543)	285 (160–423)	263 (151–419)	0.038
Onset to puncture	363 (240–541)	390 (285–620)	340 (228–515)	355 (233–502)	0.021
Onset to recanalization	448 (330–642)	475 (361–740)	433 (324–599)	435 (311–637)	0.051
Puncture to recanalization	75 (50–110)	78 (50–105)	77 (50–113)	75 (54–112)	0.984
IVT, *n* (%)	118 (25.0)	42 (26.1)	39 (25.3)	37 (23.6)	0.868
Anesthesia, *n* (%)	390 (82.6)	130 (80.7)	129 (83.8)	131 (83.4)	0.738
mTICI score 2b–3, *n* (%)	409 (86.7)	138 (85.7)	133 (86.4)	138 (87.9)	0.842

Values are presented as *n* (%) or median (interquartile range [IQR]).

^a^There were 7 patients with missing data, 3 in the Tertile 1 group, 1 in the Tertile 2 group, and the 3 in Tertile 3 group. ^b^ There were 5 patients with missing data, 3 in the Tertile 1 group, 1 in the Tertile 2 group, and the 1 in Tertile 3 group. ASITN/SIR, American Society of Interventional and Therapeutic Neuroradiology/Society of Interventional Radiology; ASPECTS, Alberta Stroke Program Early CT Score; BUN, blood urea nitrogen; CE, cardiac embolism; DBP, diastolic blood pressure; ICA, internal carotid artery; IQR, interquartile ranges; IVT, intravenous thrombolysis; LAA, large artery atherosclerosis; mTICI, modified Thrombolysis In Cerebral Infarction; NIHSS, National Institutes of Health Stroke Scale; SBP, systolic blood pressure.

## Results

### Patient characteristics

Of the 490 patients who received EVT, 472 (96.3%) had available admission BUN values and constituted the final analytical cohort for this study. The demographic and clinical characteristics of patients are presented in Table 1. The median age of included patients was 69 (IQR, 59–77) years, and 272 (57.6%) of them were male. The median baseline NIHSS score was 17 (IQR, 14–20) and the median ASPECTS was 4 (IQR, 2–5). Patients in the BUN Tertile 3 group were significantly older (*p* < 0.001). Patients in BUN Tertile 1 group had significantly longer median times from symptom onset to imaging and onset to puncture time (*p* = 0.038, *p* = 0.021, respectively). Other details are shown in Table 1, as no significant differences in the remaining characteristics were observed across the three groups.

### Clinical outcomes across BUN tertiles

The clinical outcomes were summarized in Table 2. For the primary effectiveness outcome, the proportions of patients achieving an mRS score of 0–3 at 90 days decreased across increasing BUN tertiles (75 [46.6%] in Tertile 1, 60 [39.0%] in Tertile 2, and 42 [26.8%] in Tertile 3). In the primary adjusted model (Adjusted Model 1), patients in BUN Tertile 3 had significantly lower odds of achieving an mRS score of 0–3 at 90 days than those in Tertile 1 (aOR, 0.54; 95% CI, 0.31–0.95; *p* = 0.032). In the exploratory age-excluded model, the association remained in the same direction (aOR, 0.38; 95% CI, 0.22–0.65; *p* < 0.001).

**Table 2:** Clinical outcomes.

Clinical outcomes	Events, *n* (%)/median (IQR)	Crude model		Adjusted Model 1^a^		Adjusted Model 2^b^	
		OR (95% CI)	*p* value	aOR (95% CI)	*p* value	aOR (95% CI)	*p* value
Primary effectiveness outcome							
mRS score 0–3 at 90 days, *n* (%)^c^							
Tertile 1	75 (46.6)	1 (reference)	NA	1 (reference)	NA	1 (reference)	NA
Tertile 2	60 (39.0)	0.73 (0.47–1.15)	0.172	0.79 (0.47–1.34)	0.385	0.70 (0.42–1.16)	0.162
Tertile 3	42 (26.8)	0.42 (0.26–0.67)	<0.001	0.54 (0.31–0.95)	0.032	0.38 (0.22–0.65)	<0.001
Secondary effectiveness outcomes							
mRS score at 90 days, median (IQR)^d^							
Tertile 1	4 (3–6)	1 (reference)	NA	1 (reference)	NA	1 (reference)	NA
Tertile 2	4 (3–6)	0.74 (0.50–1.10)	0.138	0.81 (0.53–1.23)	0.322	0.75 (0.49–1.13)	0.167
Tertile 3	5 (3–6)	0.54 (0.36–0.80)	0.002	0.73 (0.47–1.14)	0.167	0.58 (0.38–0.88)	0.011
mRS score 0–2 at 90 days, *n* (%)^c^							
Tertile 1	39 (24.2)	1 (reference)	NA	1 (reference)	NA	1 (reference)	NA
Tertile 2	33 (21.4)	0.85 (0.50–1.45)	0.555	0.97 (0.54–1.74)	0.924	0.85 (0.48–1.50)	0.579
Tertile 3	29 (18.5)	0.71 (0.41–1.22)	0.212	0.93 (0.50–1.72)	0.813	0.66 (0.37–1.18)	0.159
mRS score 0–4 at 90 days, *n* (%)^c^							
Tertile 1	95 (59.0)	1 (reference)	NA	1 (reference)	NA	1 (reference)	NA
Tertile 2	82 (53.2)	0.79 (0.51–1.24)	0.303	0.88 (0.52–1.50)	0.641	0.79 (0.47–1.30)	0.350
Tertile 3	67 (42.7)	0.52 (0.33–0.81)	0.004	0.72 (0.42–1.24)	0.24	0.51 (0.31–0.85)	0.010
Safety outcomes							
Mortality at 90 days, *n* (%)^c^							
Tertile 1	52 (32.3)	1 (reference)	NA	1 (reference)	NA	1 (reference)	NA
Tertile 2	63 (40.9)	1.45 (0.92–2.30)	0.113	1.38 (0.81–2.33)	0.234	1.47 (0.88–2.47)	0.141
Tertile 3	76 (48.4)	1.97 (1.25–3.10)	0.004	1.49 (0.88–2.53)	0.141	1.86 (1.12–3.10)	0.017
sICH within 48 h, *n* (%)^c^							
Tertile 1	22 (13.7)	1 (reference)	NA	1 (reference)	NA	1 (reference)	NA
Tertile 2	20 (13.0)	0.94 (0.49–1.81)	0.860	0.96 (0.49–1.89)	0.911	0.97 (0.49–1.90)	0.925
Tertile 3	17 (10.8)	0.77 (0.39–1.51)	0.442	0.69 (0.34–1.41)	0.312	0.71 (0.36–1.42)	0.336
Any ICH, *n* (%)^c^							
Tertile 1	62 (38.5)	1 (reference)	NA	1 (reference)	NA	1 (reference)	NA
Tertile 2	51 (33.1)	0.79 (0.50–1.25)	0.319	0.75 (0.46–1.20)	0.229	0.78 (0.48–1.26)	0.307
Tertile 3	59 (37.6)	0.96 (0.61–1.51)	0.864	0.90 (0.55–1.46)	0.664	0.94 (0.59–1.50)	0.786

Characteristics of the clinical outcomes are presented as *n* (%) or median (interquartile range [IQR]). ^a^Model 1 was adjusted for age, sex, baseline NIHSS score, ASPECTS score, occlusion site, stroke etiology, onset to recanalization time, and IVT. ^b^Model 2 was adjusted for all covariates in Model 1 except for age. ^c^The odds ratios were estimated from a binary logistic regression model. ^d^The common odds ratio (cOR) was estimated from an ordinal logistic regression model. A cOR greater than 1 indicates a shift toward better functional outcomes relative to the reference group. aOR, adjusted odds ratio; ASPECTS, Alberta Stroke Program Early CT Score; BUN, blood urea nitrogen; CI, confidence interval; ICH, intracranial hemorrhage; IVT, intravenous thrombolysis; mRS, modified Rankin Scale; NA, not applicable; NIHSS, National Institutes of Health Stroke Scale; OR, odds ratio; sICH, symptomatic intracranial hemorrhage.

For secondary effectiveness outcomes, the median 90-day mRS scores were 4 (IQR, 3–6), 4 (IQR, 3–6), and 5 (IQR, 3–6) in BUN Tertiles 1, 2, and 3, respectively, with the distribution of mRS scores shown in Figure 2. In the ordinal logistic regression analysis, patients in Tertile 3 showed a shift toward worse functional outcomes compared with those in Tertile 1 in the crude model (cOR, 0.54; 95% CI, 0.36–0.80; *p* = 0.002). This association was attenuated and was not statistically significant in the primary adjusted model (*p* = 0.167), whereas it remained statistically significant in the model excluded age (acOR, 0.58; 95% CI, 0.38–0.88; *p* = 0.011). In the fully adjusted ordinal logistic regression model, the Brant test showed no evidence of violation of the proportional odds assumption (*p* = 0.195). The proportions of patients achieving an mRS score of 0–4 were 59.0, 53.2, and 42.7% in Tertiles 1, 2, and 3, respectively. Although Tertile 3 was associated with lower odds of achieving an mRS score of 0–4 in the crude model (*p* = 0.004) and the exploratory age-excluded model (*p* = 0.010), the association was not statistically significant in the primary adjusted model (*p* = 0.240). Similarly, the proportions achieving functional independence (mRS score 0–2) were 24.2, 21.4, and 18.5%, respectively, and no statistically significant association between BUN tertiles and functional independence was observed.

**Figure 2:**
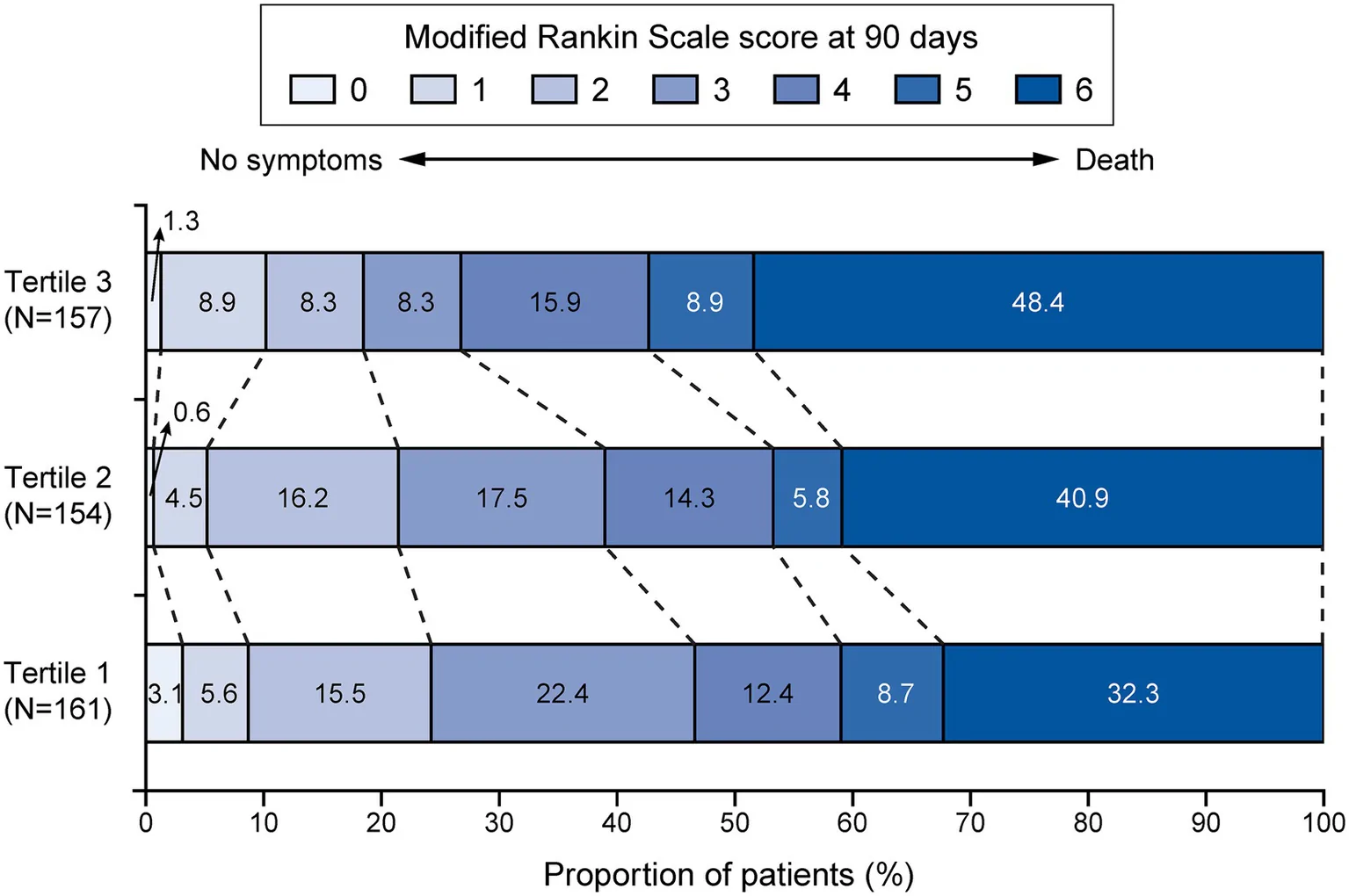
Modified Rankin Scale score distribution at 90 days. The distribution of the modified Rankin Scale scores at 90 days. Clinical outcomes at 90 days follow-up according to BUN tertiles.

Furthermore, statistical significance differences that the BUN Tertile 3 group tends to associate with a higher probability of mortality at 90 days compared with BUN Tertile 1 group can be observed in Crude Model and Adjusted Model 2 (*p* = 0.004, *p* = 0.017, respectively). No statistically significant associations between BUN tertiles and sICH within 48 h or any ICH were observed in any of the models.

Sensitivity analyses accounting for center-level clustering yielded results consistent with the primary analyses (Supplementary Table S1). After including participating center as a random intercept, patients in BUN Tertile 3 remained less likely to achieve an mRS score of 0–3 at 90 days compared with those in Tertile 1 (aOR, 0.55; 95% CI, 0.31–0.96; *p* = 0.035). The estimates for the secondary effectiveness and safety outcomes were also materially unchanged.

### Continuous and restricted cubic spline analyses

When admission BUN was modeled as a continuous variable, increasing BUN was significantly associated with a lower predicted probability of achieving an mRS score of 0–3 at 90 days (*p* = 0.005; Supplementary Figure S1A). In contrast, the associations with an mRS score of 0–2 at 90 days (*p* = 0.585), 90-day mortality (*p* = 0.059), and sICH within 48 h (*p* = 0.711) did not reach statistical significance (Supplementary Figure S1B–D). In the RCS analyses, no significant overall associations were observed for an mRS score of 0–3 (*P* for overall = 0.262), an mRS score of 0–2 (*P* for overall = 0.641), mortality (*P* for overall = 0.188), or sICH (*P* for overall = 0.624). There was also no evidence of non-linearity for any of these outcomes (*P* for non-linearity = 0.817, 0.503, 0.394, and 0.496, respectively; Supplementary Figure S2).

### Subgroup analyses

Exploratory subgroup analyses were performed to examine effect heterogeneity in the association between BUN tertiles and mRS scores of 0–3 at 90 days across subgroups. As shown in Figure 3, an interaction according to age category was observed (*P* for interaction = 0.036). Among patients aged <80 years, the OR for Tertile 3 versus Tertile 1 was 0.35 (95% CI, 0.19–0.63), whereas among patients aged ≥80 years, the corresponding OR was 1.66 (95% CI, 0.21–12.99). No significant heterogeneity was found in other subgroups.

**Figure 3:**
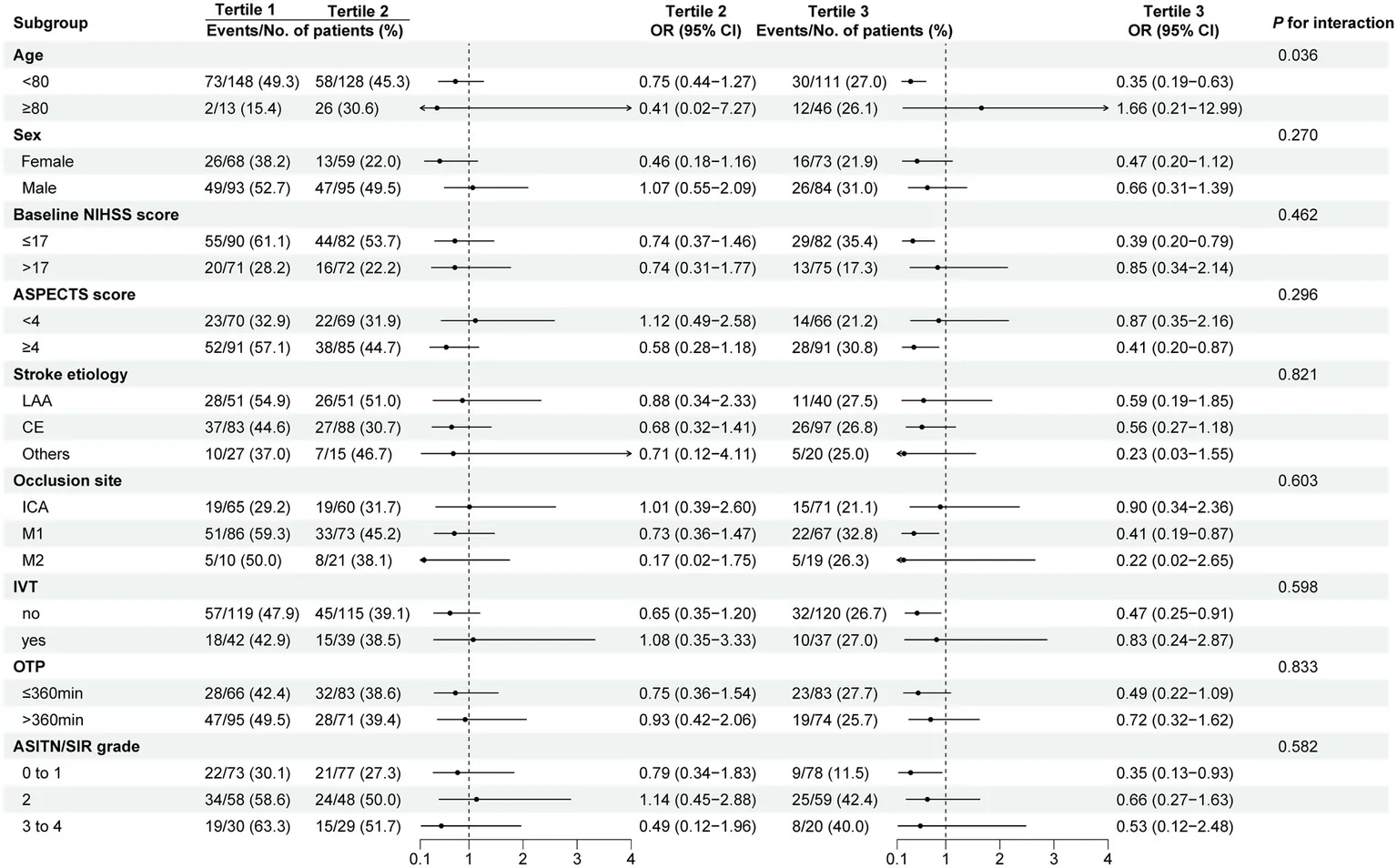
Subgroup analysis. The forest plot shows the association between BUN tertiles and the primary outcome in all subgroups. Odds ratios (OR), 95% CIs and *P* for interaction were estimated from logistic regression models. Abbreviations are as in Tables 1 and 2.

### Marginal effect analysis

In addition, we employed marginal effect analyses to investigate the association between several clinical factors (including age, ASPECTS, baseline NIHSS score, and puncture to recanalization time [PTR]) and 90-day clinical outcomes (an mRS score of 0–3 and mortality). The results were presented in Supplementary Figure S1 and S2. Our analyses demonstrated that within all groups, the predicted probability of an mRS score of 0–3 progressively decreased with increasing age, baseline NIHSS score, procedure time, and with decreasing ASPECTS (Supplementary Figure S1). The opposite trend was observed for 90-day mortality (Supplementary Figure S2). Across the groups in the illustrations, with increasing BUN, the predicted probability of favorable outcome was lower, whereas the predicted probability of mortality was higher. Nevertheless, no interaction effect was found between the selected clinical factors and admission BUN (*P* for interaction >0.05 in all models).

## Discussion

To our knowledge, this is the first study to investigate the association between admission BUN and 90-day clinical outcomes among patients with large ischemic stroke undergoing EVT. In this study, we demonstrated that admission BUN >6.80 mmol/L (BUN Tertile 3 group) was associated with a significantly lower probability of achieving favorable functional outcome at 90 days. Nevertheless, the probability of sICH within 48 h and any ICH showed no significant difference among the groups in our study.

Several previous studies have demonstrated that BUN serves as a prognostic factor for critically ill patients, and in some cases, it may be a stronger predictor than serum creatinine (SCr) and estimated glomerular filtration rate (eGFR) (9–11, 18). In particular, You S et al. (15) demonstrated that patients with BUN ≥6.70 mmol/L had a 3.75-fold higher risk of in-hospital mortality after adjusting for potential confounders including age. No statistically significant association between elevated BUN and mRS score ≥3 at discharge was found. However, in our study, patients with BUN >6.80 mmol/L had significantly lower odds of mRS score 0–3 at 90 days. Nevertheless, this discrepancy is explainable. First, our cohort consisted of patients with large infarcts exclusively, who inherently had a higher risk of ischemia-recanalization injury and cerebral edema, leading to more deteriorated neurological function (19). Second, we set 90-day mRS score 0–3 as primary effectiveness outcome, which was a more stable and reliable measurement of ultimate functional status for stroke patients compared to in-hospital mortality and mRS score ≥3 at discharge (20, 21).

Regarding mortality at 90 days, our study revealed a nuanced relationship between admission BUN and 90-day mortality (*p* = 0.017). As described previously, the association was attenuated and lost statistical significance after adjusting for age and other confounders in Adjusted Model 1. Therefore, marginal effect analysis was conducted and demonstrated no significant interaction between BUN and age for mortality (*P* for interaction = 0.891). Collectively, in our study, the mortality at 90 days increased with increasing age of patients, which is in accordance with previous studies (2, 4). Furthermore, the pattern that higher admission BUN corresponds to higher probability of mortality at 90 days may be consistent across the entire age spectrum. A plausible explanation is that elevated BUN in the context of a large ischemic stroke reflects a state of profound systemic insult (16), which may be strongly associated with mortality irrespective of the patient’s age. sICH is a severe complication following EVT for acute ischemic stroke, and is characterized by poor functional outcomes and increased mortality rates (22). In our study, no statistically significant difference was seen among the three groups. The finding suggested that although elevated BUN may damage brain tissues like white matter, it is not associated with sICH, which is consistent with previous studies (23–25). Additionally, we investigated whether other clinical features including ASPECTS, baseline NIHSS score, and PTR interacted with admission BUN tertiles. The results indicated no statistically significant interaction between these factors and admission BUN tertiles existed.

Previous studies have discovered that elevated BUN is associated with worse clinical outcomes in patients with stroke. The following hypotheses may give explanations to this phenomenon. First, elevated BUN reflects the deterioration of hemodynamic state at admission (26, 27), which exerts a detrimental influence on clinical outcomes of AIS patients and also in patients treated with EVT (28, 29). Second, BUN is a reliable indicator for assessing catabolic status, and high BUN represents a state of hypermetabolism (30, 31), which may impair recovery and contribute to higher probability of disability in patients with ischemic stroke (32). Third, severe inflammatory responses are commonly observed during the acute stage of stroke (33), which may also contribute to hypermetabolism (34). Fourth, it has been reported that in chronic kidney disease patients who are often characterized by elevated BUN, there is a tendency of more cerebral microbleeds or white matter lesions (24, 25, 35). Both factors were associated with poor clinical outcomes of ischemic stroke patients undergoing EVT (23, 36). Collectively, we could infer with caution that similar mechanisms may be involve in the context of large ischemic stroke.

More broadly, other routinely available biochemical markers have also observed to be associated with clinically relevant outcomes in acute care populations. For example, serum uric acid abnormalities have been associated with ischemic stroke in emergency department patients (37), while hypouricemia has been associated with higher 6-month mortality in a broader emergency department population (38). These observations support the potential value of inexpensive biochemical parameters, including BUN, as complementary markers in clinical risk assessment rather than stand-alone prognostic indicators.

### Strengths and limitations

The strength of this study lies in the inclusion of patients from a prospective multicenter cohort, which facilitated a reliable analysis. Furthermore, it is the first study to investigate the association between admission BUN and clinical outcomes in patients with large ischemic stroke undergoing EVT to date.

However, our study has several limitations. First, as a post-hoc analysis rather than a randomized controlled trial, it remained susceptible to unmeasured confounding, despite comprehensive statistical adjustments for a wide range of known prognostic factors. Second, we relied on a single BUN measurement at admission, which provides only a static snapshot and does not capture the dynamic changes in BUN that may occur during the hospital course. Third, an interaction between BUN tertiles and categorical age was observed in the exploratory subgroup analysis. However, this finding should be interpreted cautiously. The number of patients aged ≥80 years was limited, resulting in imprecise estimates with wide confidence intervals, and multiple subgroup comparisons were performed without adjustment for multiplicity. Moreover, the interaction was not statistically significant when age was evaluated continuously. Therefore, the apparent age-related heterogeneity should be regarded as hypothesis-generating and requires confirmation in larger studies. Fourth, several renal function-, hydration-, nutritional-, and treatment-related variables that may influence BUN levels, including serum creatinine, estimated glomerular filtration rate, the BUN/creatinine ratio, serum albumin, fluid status, diuretic use, chronic kidney disease, and acute kidney injury, were not systematically collected in the original registry and could not be adjusted for. Consequently, residual confounding by these variables cannot be excluded, and the observed association should not be interpreted as demonstrating that BUN is prognostically independent of these factors. Future prospective studies incorporating comprehensive assessment of these variables are warranted.

## Conclusion

In conclusion, in patients with large ischemic stroke undergoing EVT, an elevated admission BUN (>6.80 mmol/L) is associated with a lower likelihood of favorable functional outcome 90 days. This readily available biomarker may serve as a valuable aid for early prognostication and risk stratification in this population. However, validation is still needed in RCTs or larger cohort studies.

## Data Availability

The data analyzed in this study is subject to the following licenses/restrictions: Data are available upon reasonable request. Requests to access these datasets should be directed to wuhanu_pj@163.com.
