# Supplementary material for: Association between admission blood urea nitrogen and clinical outcomes in patients with large ischemic stroke undergoing endovascular treatment
Source: Front Neurol. 2026 Sep 23;17:1880623. doi: 10.3389/fneur.2026.1880623 (PMC13642688; doi:10.3389/fneur.2026.1880623)
Supplement: Supplementary file 1 [file Supplementary_file_1.docx]

**Supplementary materials**

**Table S1. Clinical outcomes with mixed effect model**

**Figure S1. Association between admission blood urea nitrogen and predicted probabilities of clinical outcomes**

**Figure S2. Restricted cubic spline analyses of the associations between admission blood urea nitrogen levels and clinical outcomes**

**Figure S1. Association of clinical features with the probability of mRS score 0–3 at 90 days**

**Figure S2. Association of clinical features with the probability of mortality at 90 days**

**Table S1. Sensitivity analyses of clinical outcomes using mixed effect regression models**

| **Clinical outcomes** | **Events, n (%) / median (IQR)** | **Mixed Effect Model^a^** | |
| --- | --- | --- | --- |
|  |  | **aOR (95% CI)** | ***P* value** |
| **Primary effectiveness outcome** | | | |
| mRS score 0–3 at 90 days, n (%)^b^ |  |  |  |
| Tertile 1 | 75 (46.6) | 1 (reference) | NA |
| Tertile 2 | 60 (39.0) | 0.81 (0.47–1.41) | 0.463 |
| Tertile 3 | 42 (26.8) | 0.55 (0.31–0.96) | 0.035 |
| **Secondary effectiveness outcomes** | | | |
| mRS score at 90 days, median (IQR)^c^ |  |  |  |
| Tertile 1 | 4 (3–6) | 1 (reference) | NA |
| Tertile 2 | 4 (3–6) | 0.81 (0.52–1.25) | 0.342 |
| Tertile 3 | 5 (3–6) | 0.73 (0.46–1.16) | 0.184 |
| mRS score 0–2 at 90 days, n (%)^b^ |  |  |  |
| Tertile 1 | 39 (24.2) | 1 (reference) | NA |
| Tertile 2 | 33 (21.4) | 0.97 (0.54–1.74) | 0.924 |
| Tertile 3 | 29 (18.5) | 0.93 (0.50–1.72) | 0.813 |
| mRS score 0–4 at 90 days, n (%)^b^ |  |  |  |
| Tertile 1 | 95 (59.0) | 1 (reference) | NA |
| Tertile 2 | 82 (53.2) | 0.88 (0.52–1.49) | 0.641 |
| Tertile 3 | 67 (42.7) | 0.72 (0.42–1.24) | 0.240 |
| **Safety outcomes** | | | |
| Mortality at 90 days, n (%)^b^ |  |  |  |
| Tertile 1 | 52 (32.3) | 1 (reference) | NA |
| Tertile 2 | 63 (40.9) | 1.37 (0.79–2.35) | 0.261 |
| Tertile 3 | 76 (48.4) | 1.49 (0.87–2.54) | 0.147 |
| sICH within 48 h, n (%)^b^ |  |  |  |
| Tertile 1 | 22 (13.7) | 1 (reference) | NA |
| Tertile 2 | 20 (13.0) | 0.96 (0.49–1.89) | 0.911 |
| Tertile 3 | 17 (10.8) | 0.69 (0.34–1.41) | 0.312 |
| Any ICH, n (%)^b^ |  |  |  |
| Tertile 1 | 62 (38.5) | 1 (reference) | NA |
| Tertile 2 | 51 (33.1) | 0.75 (0.46–1.22) | 0.244 |
| Tertile 3 | 59 (37.6) | 0.87 (0.53–1.44) | 0.597 |

Characteristics of the clinical outcomes are presented as n (%) or median (interquartile range [IQR]).

^a^ Mixed effects regression models adjusted for age, sex, baseline NIHSS score, ASPECTS score, occlusion site, stroke etiology, onset to recanalization time, and IVT as fixed effects, with participating center included as a random intercept to account for within-center clustering.

^b^ The odds ratios were estimated from a binary logistic regression model.

^c^ The common odds ratio (cOR) was estimated from an ordinal logistic regression model. A cOR greater than 1 indicates a shift toward better functional outcomes relative to the reference group.

Abbreviations: aOR, adjusted odds ratio; ASPECTS, Alberta Stroke Program Early CT Score; BUN, blood urea nitrogen; CI, confidence interval; ICH, intracranial hemorrhage; IVT, intravenous thrombolysis; mRS, modified Rankin Scale; NA, not applicable; NIHSS, National Institutes of Health Stroke Scale; OR, odds ratio; sICH, symptomatic intracranial hemorrhage.

**Figure S1. Association between admission blood urea nitrogen and predicted probabilities of clinical outcomes**

Marginal effect plots showing the predicted probabilities of (A) an mRS score of 0–3 at 90 days, (B) an mRS score of 0–2 at 90 days, (C) mortality at 90 days, and (D) sICH within 48 h according to admission BUN levels. BUN was modeled as a continuous variable per 0.1 mmol/L increase. Solid lines indicate the estimated probabilities, and shaded areas represent 95% CIs. P values correspond to the associations between continuous BUN and the respective outcomes.

Abbreviations: BUN, blood urea nitrogen; CI, confidence interval; mRS, modified Rankin Scale; sICH, symptomatic intracranial hemorrhage.

**Figure S2. Restricted cubic spline analyses of the associations between admission blood urea nitrogen levels and clinical outcomes**

Restricted cubic spline analyses showing the associations of admission BUN levels with (A) an mRS score of 0–3 at 90 days, (B) an mRS score of 0–2 at 90 days, (C) mortality at 90 days, and (D) sICH within 48 h. Solid lines represent the estimated ORs, and shaded areas indicate the corresponding 95% CIs. The horizontal dashed line indicates an OR of 1. The gray curves represent the distribution density of admission BUN levels. P for overall indicates the overall association between BUN and the corresponding outcome, and P for non-linearity indicates the evidence for departure from linearity.

Abbreviations: BUN, blood urea nitrogen; CI, confidence interval; mRS, modified Rankin Scale; OR, odds ratio; RCS, restricted cubic spline; sICH, symptomatic intracranial hemorrhage.

**Figure S3. Association of clinical features with the probability of mRS score 0–3 at 90 days**

The probabilities of achieving an mRS score of 0–3 based on age, ASPECTS, baseline NIHSS score and puncture to recanalization time among patients in our study are presented in A, B, C and D respectively. Solid lines indicate estimated probabilities of outcomes; shaded areas represent 95% CIs.

Abbreviations: ASPECTS, Alberta Stroke Program Early CT Score; BUN, blood urea nitrogen; CI, confidence interval; mRS, modified Rankin Scale; NIHSS, National Institutes of Health Stroke Scale; PTR, puncture to recanalization.

**Figure S4. Association of clinical features with the probability of mortality at 90 days**

The probabilities of mortality based on age, ASPECTS, baseline NIHSS score and puncture to recanalization time among patients in our study are presented in A, B, C and D respectively. Solid lines indicate estimated probabilities of outcomes; shaded areas represent 95% CIs.

Abbreviations: ASPECTS, Alberta Stroke Program Early CT Score; BUN, blood urea nitrogen; CI, confidence interval; mRS, modified Rankin Scale; NIHSS, National Institutes of Health Stroke Scale; PTR, puncture to recanalization.
